# Associations of fetal and infant growth with pubertal timing

**DOI:** 10.1136/archdischild-2024-327060

**Published:** 2025-01-29

**Authors:** Sophia Blaauwendraad, Romy Gaillard, Romy Gonçalves, Fernando Rivadeneira, Gert Dohle, Edwin Oei, Annemarie Mulders, Pauline Jansen, Vincent Jaddoe

**Affiliations:** 1 Pediatrics, Erasmus MC, Rotterdam, Netherlands; 2 The Generation R Study Group, Erasmus MC, Rotterdam, Netherlands; 3 Internal Medicine and Epidemiology, Erasmus MC, Rotterdam, Netherlands; 4 Urology, Erasmus MC, Rotterdam, Netherlands; 5 Radiology and Nuclear Medicine, Erasmus MC, Rotterdam, Netherlands; 6 Gynaecology and Obstetrics, Erasmus MC, Rotterdam, Netherlands; 7 Psychology, Education, and Child Studies, Erasmus University, Rotterdam, Netherlands; 8 Yulius Center for Child and Adolescent Psychiatry, Parnassia Group, Rotterdam, Netherlands

**Keywords:** Growth, Endocrinology, Infant Development

## Abstract

**ABSTRACT:**

**Objective:**

Impaired fetal and infant growth may cause alterations in developmental programming of the hypothalamic–pituitary–gonadal axis and subsequently pubertal development. We aimed to assess associations between fetal and infant growth and pubertal development.

**Design:**

Population-based prospective birth cohort.

**Setting:**

Rotterdam, the Netherlands.

**Patients:**

5830 singleton born children.

**Interventions:**

We estimated fetal weight in second and third trimester by ultrasound. Infant growth measures were gestational age and weight at birth and infant weight at 6, 12 and 24 months.

**Main outcome measures:**

Pubertal timing outcomes included difference between chronological and skeletal age assessed using dual-energy X-ray absorptiometry, testicular or ovarian volumes assessed using MRI at 10 years, age at menarche and Tanner staging at 13 years.

**Results:**

Among girls, 1-SD scores birth weight increase was associated with larger ovarian volume at 10 years (0.07 SD (95% CI 0.02 to 0.12) and later age at menarche (0.06 (0.02 to 0.11)). Among girls, increased infant growth was associated with an older skeletal age at 10 years (difference 2.67 (95% CI 2.26 to 3.08) months), earlier menarche (difference 0.10 (95% CI −0.14 to –0.06) years) and more advance breast and pubic hair development at 13 years (difference in Tanner stages 0.09 (0.05 to 0.13) and 0.07 (0.03 to 0.12)). In boys, increased infant growth was associated with an older skeletal age (3.13 (95% CI 2.58 to 3.69) months) and a larger testicular volume (0.07 (95% 0.02 to 0.12) SD) at 10 years, and with more advance pubic hair development (0.09 (95% CI 0.05 to 0.14) at 13 years).

**Conclusion:**

Birth anthropometrics and early-life growth patterns are associated with altered pubertal development in a sex-specific manner.

**WHAT IS ALREADY KNOWN ON THIS TOPIC**

- The hypothalamic–hypopituitary–gonadal axis develops till 1-year postpartum, thus decelerated and accelerated early-life growth might cause altered pubertal development. Findings from previous studies are inconsistent but indicate sex-specific associations of early-life growth with pubertal development.

**WHAT THIS STUDY ADDS**

- This study adds to elucidate mechanisms underlying the secular trend of earlier pubertal timing by investigating associations of fetal and infant growth patterns combined with novel markers of puberty such as gonadal imaging.

**HOW THIS STUDY MIGHT AFFECT RESEARCH, PRACTICE OR POLICY**

- Further elucidation of mechanisms of earlier puberty aids in risk prediction of and potential intervention on abnormal pubertal development.

## INTRODUCTION

 Earlier puberty is associated with adverse long-term health outcomes.^1^ The hypothalamic–pituitary–gonadal axis, which controls the onset of puberty, develops in early pregnancy and remains active the first year of life.^2^ Hereafter, it enters a dormant state until its reactivation at the start of puberty.^2^ Both decelerated and accelerated fetal and infant growth may be associated with hypothalamic–pituitary–gonadal axis alterations and subsequent altered pubertal development. Previous studies on associations of fetal and infant growth show inconsistent and sex-specific results.^3^,^13^ Also, studies have been predominantly performed among girls. Additionally, no previous studies on early-life growth patterns and pubertal development included fetal growth patterns. We aimed to further elucidate the role of early-life growth in altered pubertal development.

In a population-based multiethnic cohort among 5830 mother–child pairs, we assessed the associations of fetal and infant growth patterns with markers of pubertal development among boys and girls. These included skeletal age, ovarian and testicular volumes at age 10 years, age at menarche and Tanner stages at age 13 years.

## Methods

### Study design and population

This study was embedded in the Generation R Study, a population-based prospective cohort study from fetal life onwards in Rotterdam, the Netherlands.^14^ Pregnant women who delivered between April 2002 and January 2006 were eligible for participation. Written informed consent was obtained from all mothers. Information on fetal or infant growth was available for 9472 singleton live-born children. Of those, 5362 children had information on skeletal age at 10 years, 2884 children had an MRI of the gonads at 10 years, and 3354 children had information on pubertal development at 13 years (flowchart is shown in online supplemental figure S1).

### Fetal and infant growth measures

Fetal growth ultrasound examinations were performed in mid (20.7 weeks gestational age (±SD 1.2)) and late (30.5 weeks (±SD1.1)) pregnancy.^14^ We measured fetal head and abdominal circumference, and femur length using standardised ultrasonography procedures.^14 15^ We calculated gestational age-adjusted estimated fetal weight using the formula of Hadlock and created SD scores (SDS).^16^ Information on child sex, gestational age at birth and birth weight was obtained from medical records. We calculated gestational age and sex-adjusted size at birth SDS according to Niklasson growth charts.^17^

Infant weight was measured at 6, 12 and 24 months. We created age and sex-adjusted SDS using Dutch reference growth charts (Growth Analyzer BV, V.4.0).^14^ Infant weight change was defined as SDS change from birth to 24 months (3867 of 4864 children), or if unavailable at 11 months (805 children) or 6 months (192 children).

### Markers of pubertal development

Measurements of pubertal development outcomes are described in detail in online supplemental methods. At 10 years, the skeletal age was obtained from DXA-scans of the left hand by trained research nurses using an iDXA densitometer (General Electric, formerly Lunar, Madison, Wisconsin).^18^ We calculated the relative skeletal age in months by subtracting the skeletal age from the chronological age. A higher skeletal age than chronological age reflects a faster skeletal maturation, associated with earlier pubertal development.^19 20^

Testicular and ovarian volumes were measured by MRI.^14 21^ Larger testicular volume represents more advanced pubertal development, larger ovarian volume is associated with more advanced growth.^22^ Mean volumes of right and left testicles or ovaries were square root transformed to obtain normal distribution and SDS were calculated.

At 13 years, when children are usually in the midst of puberty and thus differences in pubertal development are apparent, we obtained self-reported age at menarche and pubertal development through questionnaires. Tanner staging is a common clinical marker of pubertal development using a 5-point categorical scale.^21 23^ We obtained stage of testicular, breast and pubic hair development.

### Covariates

Information on maternal age, education, parity, folic acid supplementation, alcohol use and smoking was obtained through questionnaires. Information on ethnicity was obtained, and participants were categorised as Dutch, European (Turkish, European) or non-European (Indonesian, Cape Verdean, Moroccan, Dutch Antilles, Surinamese, African, American western/non-western, Asian western/non-western, Oceanian). In mothers at enrolment, and in children aged 10 and 13 years, weight was measured once using a Seca 888 scale and height was measured once using a Harpender stadiometer, Holtain Limited, which are both accurate to one decimal place.^14^ Maternal prepregnancy and early pregnancy and child’s age-adjusted body mass index were calculated as weight (kilograms) divided by height (metres) squared.^24^

### Statistical analysis

First, to identify potential non-response bias, we compared the characteristics of mother–child pairs with outcome data available in our study to mother–child pairs without outcome data available. Second, we calculated pairwise Pearson’s correlation coefficients between the pubertal development outcomes.^25^ Third, we assessed the associations of gestational age, weight and size for gestational age at birth with pubertal development outcomes using linear regression models. Our primary models included continuous birth characteristics as exposure, including gestational age at birth, birth weight and size for gestational age at birth. In our secondary models, we categorised these into preterm (<37 weeks) or term birth, low (<2500 g), high (>4000 g) or normal birth weight and small (<10th percentile), large (>90th percentile) or appropriate for gestational age. Additionally, we assessed the association of infant growth with pubertal development outcomes using linear regression models. We checked for the interaction with birth weight category (small for gestational age (SGA), average for gestational age (AGA), large for gestational age (LGA)), using a p value threshold of 0.10, and observed no interaction for any of the pubertal development outcomes. All analyses were first adjusted for child’s sex and age at outcome measurement (basic model), and for potential maternal age, body mass index, educational level, ethnicity, folic acid supplementation use, smoking and alcohol use in the adjusted model. These confounders were selected from previous literature and depicted in a directed acyclic graph (online supplemental figure S2). In our models on gonadal volume, we additionally adjusted for child height.^26^ As pubertal development is dependent on ethnicity, we performed sensitivity analyses including Dutch children only.^19^ We performed all analyses for girls and boys. We tested for linearity of associations and identified no non-linearity. Missing values were imputed using multiple imputation by the fully conditional specification method, and pooled results from 25 imputed datasets were reported. All statistical tests were two sided. To correct for multiple hypothesis testing, we considered false discovery rate adjusted p values of <0.05 statistically significant. Analyses were performed using R Statistical Software (V.4.2.2; R Development Core Team).

## Results

### Participant characteristics

The mean maternal age in our study sample was 30.9 years, and most mothers were highly educated, nulliparous, had a normal prepregnancy body mass index (BMI), never smoked and never drank alcohol during pregnancy (table 1, online supplemental tables S1 and S2). In children, the correlation between the pubertal development outcomes was in general low (table 2, online supplemental figure S3).

**Table 1:** General characteristics of the study population, stratified for girls and boys

	Girls (n=2992)	Boys (n=2838)
Maternal characteristics		
Maternal age in years, mean (±SD)	30.8 (5.0)	31.0 (5.0)
Highest education finished, number (%)		
Primary	252 (8.4)	249 (8.8)
Secondary	1297 (43.3)	1179 (41.5)
Higher	1443 (48.2)	1410 (49.7)
Ethnicity, n (%)*		
Dutch	1695 (56.7)	1616 (56.9)
European	439 (14.7)	401 (14.1)
Non-European	858 (28.7)	821 (28.9)
Parity, number (%)		
Nullipara	1740 (58.2)	1571 (55.4)
Multipara	1252 (41.8)	1267 (44.6)
Pre-pregnancy body mass index in kg/m^2^, median (95% range)	22.9 (18.8 to 32.6)	22.7 (18.7 to 32.4)
Smoking, number (%)		
Never smoked during pregnancy	2300 (76.9)	2164 (76.3)
Smoked until pregnancy was known	256 (8.6)	227 (8.0)
Continued smoking in pregnancy	436 (14.6)	447 (15.8)
Alcohol use, number (%)		
Never alcohol in pregnancy	1345 (45.0)	1195 (42.1)
Alcohol until pregnancy was known	394 (13.2)	424 (14.9)
Alcohol continued in pregnancy	1253 (41.9)	1219 (43.0)
Fetal characteristics		
Second trimester		
Gestational age, weeks, mean (±SD)	20.6 (1.1)	20.7 (1.2)
Estimated fetal weight, g, median (95% range)	360 (264 to 551)	367 (266 to 575)
Third trimester		
Gestational age, weeks, mean (±SD)	30.5 (1.1)	30.5 (1.1)
Estimated fetal weight, g, median (95% range)	1592 (1249 to 2062)	1631 (1266 to 2094)
Birth and infant characteristics		
Gestational age at birth, weeks, median (95% range)	40.1 (37.0 to 42.0)	40.1 (37.0 to 42.1)
Birth weight, grams, mean (±SD)	3367.0 (535)	3503 (563)
Ethnicity, n (%)*		
Dutch	1793 (59.9)	1674 (59.0)
European	416 (13.9)	392 (13.8)
Non-European	783 (26.2)	772 (27.2)
6-month visit		
Age, months, mean (±SD)	6.3 (0.6)	6.2 (0.7)
Weight, kg, mean (±SD)	7.6 (0.8)	8.2 (0.9)
Height, cm, mean (±SD)	66.7 (2.5)	68.5 (2.6)
11-month visit		
Age, months, mean (±SD)	11.1 (0.6)	11.1 (0.6)
Weight, kg, mean (±SD)	9.3 (1.0)	9.9 (1.0)
Height, cm, mean (±SD)	73.6 (2.5)	75.1 (2.5)
24-month visit		
Age, months, mean (±SD)	25.1 (1.2)	25.1 (1.2)
Weight, kg, mean (±SD)	12.7 (1.5)	13.2 (1.5)
Height, cm, mean (±SD)	87.7 (3.4)	88.8 (3.3)
Child characteristics		
10-year visit		
Age, months, mean (±SD)	9.7 (0.3)	9.7 (0.4)
Body mass index, kg/m^2^, median (95% range)	17.1 (14.3 to 23.7)	16.9 (14.4 to 22.8)
13-year visit		
Age, months, mean (±SD)	13.6 (0.4)	13.5 (0.4)
Body mass index, kg/m^2^, median (95% range)	19.5 (16.1 to 29.2)	18.6 (15.7 to 26.6)

Values presented as mean (± SD, median (95% range)) or number of participants (valid %). Missing values of covariates were imputed using multiple imputation by the fully conditional specification method.

Non-European consists of Indonesian, Cape Verdean, Moroccan, Dutch Antilles, Surinamese, African, American western, American non-western, Asian western, Asian non-western, Oceanian.

* European consists of Turkish and European.

**Table 2:** Child outcome characteristics

	Girls	Boys
10-year visit		
Relative skeletal age in months, median (95% range)*	−1.4 (−21.5, 16.4)	−7.0 (−32.2, 16.3)
Ovarian volume in cm^3^, median (95% range)	1.5 (0.4, 4.1)	NA
Testicular volume in cm^3^, median (95% range)	NA	1.1 (0.6, 3.1)
13-year visit		
Genital Tanner stage, n (%)		
Stage 1 (prepubertal)	NA	26 (1.8)
Stage 2	NA	218 (15.5)
Stage 3	NA	585 (41.6)
Stage 4	NA	475 (33.8)
Stage 5 (adult)	NA	101 (7.2)
Breast Tanner stage, n (%)		
Stage 1 (prepubertal)	20 (1.1)	NA
Stage 2	210 (11.2)	NA
Stage 3	749 (40.0)	NA
Stage 4	733 (39.1)	NA
Stage 5 (adult)	162 (8.6)	NA
Pubic hair Tanner stage, n (%)		
Stage 1 (prepubertal)	23 (1.4)	71 (4.4)
Stage 2	150 (8.8)	299 (18.5)
Stage 3	465 (27.4)	571 (35.2)
Stage 4	710 (41.8)	578 (35.7)
Stage 5 (adult)	350 (20.6)	101 (6.2)
Age at menarche, years, mean (±SD)	12.4 (0.9)	NA

Values presented as mean (± SD, median (95% range) or number of participants (valid %).

* Relative skeletal age is calculated by subtracting the chronological age from the skeletal age.

### Birth outcomes and pubertal timing

Among girls, with adjustment for covariates, 1-SDS higher sex-adjusted and gestational age-adjusted size at birth was associated with 0.07 SD (95% CI 0.02 to 0.12) increase in ovarian volume at 10 years and 0.06 (0.02, 0.11) years older age at menarche (table 3). Similar associations were present in the basic model (online supplemental table S3). Additionally, in girls, preterm birth was associated with lower breast development stages (difference −0.25 (95% CI −0.43 to –0.06) for girls born preterm as compared with girls born term) (table 4, basic model, online supplemental table S4). Among boys, birth characteristics were not associated with pubertal development outcomes.

**Table 3:** Association of birth characteristics with bone age, ovarian/testicular volume at 10 years, and age at menarche, adjusted model

Birth outcomes	Girls			Boys	
	Difference in relative bone age, months (95% CI)	SDS difference in ovarian volume (95% CI)	Difference in age at menarche, years (95% CI)	Difference in relative bone age, months (95% CI)	SDS difference in testicular volume (95% CI)
Gestational age at birth					
Gestational age continuously (per week)†	−0.20 (−0.44, 0.04)	0.00 (−0.03, 0.03)	0.01 (−0.02, 0.03)	−0.12 (−0.44, 0.19)	0.02 (−0.02, 0.04)
Preterm birth‡	0.61 (−1.36, 2.58)	−0.01 (−0.23, 0.24)	−0.04 (−0.23, 0.14)	0.51 (−2.15, 3.17)	0.03 (−0.21, 0.27)
Term birth	Reference	Reference	Reference	Reference	Reference
Birth weight					
Birth weight continuously (per SD)†	−0.02 (−0.46, 0.43)	0.00 (−0.05, 0.05)	0.05 (0.01, 0.10)	0.31 (−0.24, 0.86)	0.02 (−0.03, 0.07)
<2500 g‡	0.32 (−1.72, 2.35)	−0.02 (−0.23, 0.20)	−0.11 (−0.29, 0.06)	2.05 (−0.78, 4.88)	−0.02 (−0.25, 0.29)
2500–4000 g	Reference	Reference	Reference	Reference	Reference
>4000 g‡	0.87 (−0.55, 2.29)	0.03 (−0.20, 0.14)	0.08 (−0.04, 0.21)	1.31 (−0.20, 2.81)	0.13 (−0.01, 0.27)
Size for gestational age at birth					
Sex and gestational age adjusted size at birth (per SD)†	−0.09 (−0.35, 0.52)	0.07 (0.02, 0.12)*	0.06 (0.02, 0.11)*	0.57 (0.01, 1.13)	0.04 (−0.02, 0.09)
Small (<10th percentile)‡	0.60 (−0.93, 2.13)	−0.01 (−0.19, 0.17)	−0.10 (−0.24, 0.05)	1.55 (−0.69, 3.79)	0.09 (−0.11, 0.30)
Appropriate (10–90th percentile)	Reference	Reference	Reference	Reference	Reference
Large (>90th percentile)‡	0.76 (−0.82, 2.33)	−0.02 (−0.20, 0.17)	0.11 (−0.03, 0.25)	0.91 (−0.71, 2.53)	0.14 (−0.01, 0.29)

Models include maternal age, body-mass index, educational level, ethnicity, folic acid supplementation use, smoking and alcohol use in pregnancy and child age at outcome measurement (except for relative bone age).

* Statistically significant (False Discovery Rate (FDR) adjusted p value<0.05).

† Values represent the change (95% CI) in relative bone age (months), ovarian/testicular volume (SD) or age at menarche (years) per unit increase.

‡ Values represent the change (95% CI) in relative bone age (months), ovarian/testicular volume (SD) or age at menarche (years) for this birth weight category as compared with the reference category.

SDS, SD scores.

**Table 4:** Associations of birth characteristics with pubertal Tanner stage at 13 years, adjusted model

Birth outcome	Girls		Boys	
	Difference in breast development stage (95% CI)	Difference in pubic hair development stage (95% CI)	Difference in genital development stage (95% CI)	Difference in pubic hair development stage (95% CI)
Gestational age at birth				
Gestational age continuously (per week)†	0.02 (0.01, 0.05)	−0.03 (−0.06, 0.00)	−0.01 (−0.03, 0.02)	−0.02 (−0.04, 0.00)
Preterm birth	−0.25 (−0.42, 0.06)*	−0.01 (−0.22, 0.20)	0.11 (−0.10, 0.30)	0.07 (−0.14, 0.28)
Term birth	Reference	Reference	Reference	Reference
Birth weight				
Birth weight continuously (per SD)†	0.00 (−0.04, 0.04)	−0.05 (−0.10, 0.00)	−0.01 (−0.06, 0.04)	−0.05 (−0.08, 0.00)
<2500 g‡	0.02 (−0.16, 0.21)	0.27 (0.04, 0.51)	0.06 (−0.18, 0.27)	0.18 (−0.05, 0.40)
2500–4000 g	Reference	Reference	Reference	Reference
>4000 g‡	0.01 (−0.11, 0.13)	0.03 (−0.11, 0.18)	−0.05 (−0.17, 0.07)	−0.04 (−0.16, 0.08)
Size for gestational age at birth				
Sex and gestational age adjusted size at birth (per SD)†	−0.04 (−0.08, 0.00)	−0.03 (−0.08, 0.01)	0.00 (−0.05, 0.05)	−0.03 (−0.07, 0.01)
Small (<10th percentile)‡	0.01 (−0.13, 0.15)	0.10 (−0.07, 0.28)	0.05 (−0.13, 0.23)	0.16 (−0.02, 0.34)
Appropriate (10–90th percentile)	Reference	Reference	Reference	Reference
Large (>90th percentile)‡	−0.02 (−0.15, 0.12)	−0.01 (−0.17, 0.15)	−0.07 (−0.20, 0.05)	−0.04 (−0.16, 0.09)

Models include maternal age, body mass index, educational level, ethnicity, folic acid supplementation use, smoking and alcohol use in pregnancy and child age at outcome measurement.

* Statistically significant (False Discovery Rate (FDR) adjusted p value<0.05).

† Values represent change (95% CI) in Tanner stage per unit increase.

‡ Values represent change (95% CI) in Tanner stage for this birth weight category as compared with the reference category.

### Fetal and infant growth and pubertal timing

In girls, increased infant growth between birth and 24 months was associated with a higher relative bone age at 10 years (difference 2.67 (95% CI 2.26 to 3.08) months per SD increase in infant growth) (table 5). Additionally, increased infant growth was associated with an earlier age at menarche (difference 0.10 (95% CI −0.14 to –0.06) years). Similar associations were present in the basic model (online supplemental table 5). Also, increased infant growth was associated with more advanced breast and pubic hair development (difference in Tanner stages 0.09 (0.05 to 0.13) and 0.07 (0.03 to 0.12), respectively) (online supplemental tables S6 and S7). Likewise, in boys, increased infant growth was associated with a higher relative bone age and a larger testicular volume at 10 years (differences in bone development 3.13 (95% CI 2.58 to 3.69) months and in testicular volume 0.07 (95% CI 0.02 to 0.12) SD). In line with this, increased infant growth was associated with more advance pubic hair development in boys (difference in Tanner stages 0.09 (95% CI 0.05 to 0.14)).

**Table 5:** Association of infant growth with bone age, ovarian/testicular volume at 10 years and age at menarche, adjusted model

Girls			Boys	
Difference in relative bone age, months (95% CI)	SDS difference in ovarian volume (95% CI)	Difference in age at menarche, years (95% CI)	Difference in relative bone age, months (95% CI)	SDS difference in testicular volume (95% CI)
2.67 (2.26, 3.08)*	0.05 (0.00, 0.10)*	−0.10 (−0.14 to 0.06)*	3.13 (2.58, 3.69)*	0.07 (0.02, 0.12)*

Models include maternal age, body mass index, educational level, ethnicity, folic acid supplementation use, smoking and alcohol use in pregnancy and child age at outcome measurement (except for relative bone age).

* Statistically significant (False Discovery Rate (FDR) adjusted p value<0.05). Values represent the change (95% CI) in relative bone age (months), testicular/ovarian volume (SD) or age at menarche (years) per SD increase in infant growth. Relative bone age was calculated by subtracting the skeletal age from the chronological age.

SDS, SD scores.

### Sensitivity analysis

Our sensitivity analysis including Dutch children only in general showed similar associations as the main analysis (online supplemental tables S8–S11).

## Discussion

In this population-based prospective cohort study among 5830 children, in girls, we observed associations of higher birth weight with larger ovarian volume at 10 years and later menarche. Preterm birth was associated with later breast development. In boys, no associations of birth outcomes with pubertal development were present. In both sexes, increased infant growth was associated with an older bone age at 10 years. Additionally, increased infant growth was associated with earlier menarche, breast development and pubic hair development in girls, and a larger testicular volume and pubic hair development in boys. These associations were independent of fetal growth.

### Interpretation of the main findings

Over the last decades, age at pubertal onset has decreased in girls and to the lesser extent in boys.^27 28^ Early-life exposure to an adverse environment might alter fetal and infant development patterns.^1 29^ Previous studies suggest that accelerated and decelerated early-life growth might influence the development of hormonal axes and subsequently pubertal development.^30 31^

Previous literature on associations of birth characteristics with pubertal development is inconsistent. A meta-analysis reported that low birth weight or size among 450 girls, but not among 390 boys, was associated with earlier pubertal onset and menarche as compared with normal birth weight.^3^ However, heterogeneity was high and different definitions of pubertal were applied. Similar associations were reported among 8068 Danish girls.^9^ In contrast, two large studies from the UK and Hongkong reported no associations of birth anthropometrics with pubertal onset.^4 5^ Studies among smaller samples were inconsistent.^6^,^832 33^ In the current study, we observed that higher birth weight was associated with a later age at menarche and later breast development. In contrast with the previous literature, this is indicative of a later onset of puberty. Differences between our and previous results could arise from diverse study populations or the moment of outcome assessment, as previous studies largely assessed the age of entry into puberty.

On early-life growth patterns and pubertal development, studies have predominantly been performed among girls and focused on infant growth. Three large Western cohorts including 3703 to 15 196 girls, and multiple smaller studies, reported associations of accelerated infant growth with earlier menarche, thelarche and pubarche.^59 10 12 13 32^,^34^ Among boys, literature is scarce and inconclusive.^9 11 32 33^ None of the previous studies on early-life growth patterns and pubertal development included fetal growth. In the current study, we tested for interaction of infant growth with fetal growth categories. Independent of fetal growth, increased infant growth was associated with earlier skeletal maturation and earlier pubic hair development in both boys and girls, and additionally with earlier menarche and breast development in girls. The current study adds important information by taking fetal growth into account.^35^ Also, it adds information on the effects in boys and is in line with previous studies among girls.

The association of low birth weight with earlier pubertal development might be explained by a compensatory mechanism, in which impaired development of hormonal axes in early life results in a ‘catch-up growth’ causing earlier pubertal maturation. Associations of birth outcomes with pubertal development were mainly present in girls. Further elucidating underlying potential sex-specific endocrine hormone processes by measuring sex-steroid levels throughout puberty might be of interest.

At 10 years, higher birth weight was associated with a larger ovarian volume, which is not in line with the previous literature.^36 37^ Ovarian volume based on antral follicle count might represent the follicle pool established in the fetus rather than the phase of pubertal development. Future studies should focus on antral follicle count, ovarian architecture, long-term outcomes of fertility and ovarian reserve and possibly steroid levels, which are useful in elucidating underlying physiology related to ovarian size, such as onset of puberty or polycystic ovarian syndrome. Previous literature has shown that pubertal development is dependent on ethnicity.^38^ In general, this did not affect our associations.

The current study suggests that increased infant growth is associated with earlier skeletal maturation. Skeletal maturation is a complex hormonal process, is an accurate indicator of biological maturity and is predictive of pubertal development. Indeed, associations with pubertal development were present. Our results suggest that infancy is an important time window in the development of hormonal axes, and thus of higher susceptibility to adverse environmental exposures.

### Methodological considerations

The most important strength of our study is the prospective design, allowing repeated measurements of fetal and infant growth and multiple pubertal development outcomes. We included a large population-based sample. Non-response might have led to selection of a healthier population. Although we observed high reproducibility of our fetal ultrasound measurements, these still may be liable to measurement errors.^15^ Despite the known relation of skeletal maturation and pubertal development, correlation between skeletal bone age and other markers of pubertal development such as Tanner staging were low. Skeletal bone age might be more predictive for timing of pubertal onset rather than tempo of pubertal development. The sample size of the outcome skeletal bone age was almost two times as large as the other pubertal development outcomes, which might have led to more power to identify associations. Also, self-assessment of Tanner staging might induce misclassification bias and regression towards the mean. We subcategorised ethnicity categories that existed of many different subgroups. Due to lack of power of these subgroups, we were unable to further explore the role of ethnicity. Alcohol and smoking were obtained through questionnaires, which might have led to under-reporting. We were unable to control for parental pubertal data as we had no information on this, potentially biasing our results. Due to the observational nature of the study, differences in unmeasured characteristics between participants might have confounded our associations

## Conclusion

In our population-based prospective cohort study, birth anthropometrics and early-life growth patterns were related to pubertal development. Among girls, older gestational age and higher weight at birth were generally associated with later pubertal development. Among both sexes, infant growth deceleration was associated with younger bone age, whereas infant growth acceleration was associated with older bone age at 10 years. Further studies are needed to investigate potential underlying mechanisms.

## Supplementary material

10.1136/archdischild-2024-327060 online supplemental file 1

## Data Availability

Data are available upon reasonable request.
