# Supplementary material for: Associations of fetal and infant growth with pubertal timing
Source: Arch Dis Child. 2025 Jan 29;110(7):e327060. doi: 10.1136/archdischild-2024-327060 (PMC12229080; doi:10.1136/archdischild-2024-327060)
Supplement: online supplemental file 1 [file archdischild-110-7-s001.pdf]

Supplementary Material

Associations of Fetal and Infant Growth with Pubertal Timing

Sophia M Blaauwendraad, Romy Gaillard, Romy Gonçalves, Fernando Rivadeneira, Gert Dohle,

Edwin HG Oei, Annemarie GMGJ Mulders, Pauline Jansen, Vincent WV Jaddoe

Table S1. General characteristics of the study population, stratified for girls and boys. Non imputed data.

Table S2. Non-response analysis of the study population, stratified for boys and girls.

Table S3. Association of birth characteristics with bone health, ovarian/testicular volume at 10 years, and age at menarche, basic model

Table S4. Association of birth characteristics with pubertal Tanner stage at 13 years, basic model

Table S5. Association of infant growth with bone health, ovarian/testicular volume at 10 years and age at menarche, basic model.

Table S6. Association of infant growth patterns with pubertal Tanner stage at 13 years, basic model.

Table S7. Association of infant growth patterns with pubertal Tanner stage at 13 years, adjusted model.

Table S8. Association of birth characteristics with bone health, ovarian/testicular volume at 10 years, and age at menarche, adjusted model. Sensitivity analysis including Dutch children only.

Table S9. Association of birth characteristics with pubertal Tanner stage at 13 years, adjusted model. Sensitivity analysis including Dutch children only.

Table S10. Association of infant growth with bone health, ovarian/testicular volume at 10 years and age at menarche, adjusted model. Sensitivity analysis including Dutch children only.

Table S11. Association of infant growth patterns with pubertal Tanner stage at 13 years, adjusted model. Sensitivity analysis including Dutch children only.

Figure S1. Flowchart of participants included in the study.

Figure S2. Directed Acyclic Graph.

Figure S3. Plot on the correlation between the pubertal development outcomes for A) girls and B) boys

Table S1. General characteristics of the study population, stratified for girls and boys. Non imputed data.

	**Girls (n=2992)**	**Boys (n=2838)**
**Maternal characteristics**		
Maternal age in years, mean (±SD)	30.8 (5.0)	31.0 (5.0)
Highest education finished, No. (%)		
Primary	217 (7.9)	204 (7.8)
Secondary	1189 (43.1)	1075 (41.3)
Higher	1354 (49.1)	1327 (50.9)
Parity, No. (%)		
Nullipara	1691 (58.1)	1532 (55.9)
Multipara	1220 (41.9)	1209 (44.1)
Pre-pregnancy body mass index in kg/m^2, median (95% range)	22.9 (18.8, 32.6)	22.7 (18.7, 32.4)
Smoking, No. (%)		
Never smoked during pregnancy	2036 (77.4)	1868 (75.7)
Smoked until pregnancy was known	231 (8.8)	202 (8.2)
Continued smoking in pregnancy	364 (13.8)	399 (16.2)
Alcohol use, No. (%)		
Never alcohol in pregnancy	1089 (44.9)	929 (41.3)
Alcohol until pregnancy was known	324 (13.4)	327 (14.6)
Alcohol continued in pregnancy	1013 (41.8)	991 (44.1)
**Fetal characteristics**		
Second trimester		
Gestational age, weeks, mean (±SD)	20.6 (1.1)	20.7 (1.2)
Estimated fetal weight, g, median (95% range)	360 (264, 551)	367 (266, 575)
Third trimester		
Gestational age, weeks, mean (±SD)	30.5 (1.1)	30.5 (1.1)
Estimated fetal weight, g, median (95% range)	1,592 (1249, 2062)	1631 (1266, 2094)
**Birth and infant characteristics**		
Gestational age at birth, weeks, median (95% range)	40.1 (37.0, 42.0)	40.1 (37.0, 42.1)
Birth weight, grams, mean (±SD)	3367.0 (535)	3503 (563)
Ethnicity, No. (%)		
Dutch	1748 (59.9)	1647 (59.4)
European	406 (13.9)	378 (13.6)

Non-European	762 (26.1)	748 (27.0)
6 month visit		
Age, months, mean (±SD)	6.3 (0.6)	6.2 (0.7)
Weight, kg, mean (±SD)	7.6 (0.8)	8.2 (0.9)
Height, cm, mean (±SD)	66.7 (2.5)	68.5 (2.6)
11 month visit		
Age, months, mean (±SD)	11.1 (0.6)	11.1 (0.6)
Weight, kg, mean (±SD)	9.3 (1.0)	9.9 (1.0)
Height, cm, mean (±SD)	73.6 (2.5)	75.1 (2.5)
24 month visit		
Age, months, mean (±SD)	25.1 (1.2)	25.1 (1.2)
Weight, kg, mean (±SD)	12.7 (1.5)	13.2 (1.5)
Height, cm, mean (±SD)	87.7 (3.4)	88.8 (3.3)
**Child characteristics**		
10 year visit		
Age, months, mean (±SD)	9.7 (0.3)	9.7 (0.4)
Weight, kg, mean (±SD)	35.7 (7.9)	35.3 (6.8)
Height, cm, mean (±SD)	141.1 (6.9)	141.7 (6.4)
Body mass index, kg/m^2, median (95% range)	17.1 (14.3, 23.5)	16.0 (14.4, 22.8)
13 year visit		
Age, months, mean (±SD)	13.6 (0.4)	13.5 (0.4)
Weight, kg, mean (±SD)	54.6 (11.1)	53.3 (11.6)
Height, cm, mean (±SD)	163.4 (7.0)	165.3 (8.7)
Body mass index, kg/m^2, median (95% range)	19.6 (15.9, 27.4)	18.7 (15.6, 25.8)

Values presented as mean (± standard deviation (SD), median (95% range) or number of participants (valid %). Number of missing values maternal characteristics, No. (%): maternal ethnicity 147 (2.5), education 464 (8.0), parity 178 (3.1), body-mass index 444 (7.6), smoking 730 (12.5), alcohol use 1167 (19.8), child ethnicity 141 (24.2).

Values are derived from the original population without imputation of covariates.

Table S2. Non-response analysis of the study population, stratified for boys and girls.

	Girls			Boys		
**Maternal characteristics**	**Study population (n=2992)**	**Non-response population (n=1699)**	**P-value**	**Study population (n=2838)**	**Non-response population (n=1973)**	**P-value**
Maternal age in years, mean (±SD)	30.8 (5.0)	28.1 (5.5)	<0.001*	31.0 (5.0)	28.7 (5.6)	<0.001*
Ethnicity, No. (%)			<0.001*			<0.001*
Dutch	1654 (56.8)	528 (36.6)		1585 (57.2)	660 (38.4)	
European	425 (14.6)	321 (22.3)		385 (13.9)	348 (20.3)	
Non-European	834 (28.6)	592 (41.1)		800 (28.9)	710 (41.3)	
Highest education finished, No. (%)			<0.001*			<0.001*
Primary	217 (7.9)	264 (19.5)		204 (7.8)	254 (15.8)	
Secondary	1189 (43.1)	700 (51.6)		1075 (41.3)	867 (53.9)	
Higher	1354 (49.1)	392 (28.9)		1327 (50.9)	487 (30.3)	
Parity, No. (%)			<0.001*			0.071
Nullipara	1691 (58.1)	820 (51.0)		1532 (55.9)	1002 (53.2)	
Multipara	1220 (41.9)	787 (49.0)		1209 (44.1)	883 (46.8)	
Pre-pregnancy body mass index in kg/m^2, median (95% range)	22.9 (18.8, 32.6)	23.1 (18.7, 33.1)	0.132	22.7 (18.7, 32.4)	22.9 (18.5, 33.1)	0.052
Smoking, No. (%)			<0.001*			<0.001*
Never smoked during pregnancy	2036 (77.4)	925 (69.2)		1868 (75.7)	1065 (67.3)	
Smoked until pregnancy was known	231 (8.8)	119 (8.9)		202 (8.2)	128 (8.1)	
Continued smoking in pregnancy	364 (13.8)	293 (21.9)		399 (16.2)	390 (24.6)	

Alcohol use, No. (%)			<0.001*			<0.001*
Never alcohol in pregnancy	1089 (44.9)	776 (61.3)		929 (41.3)	903 (60.5)	
Alcohol until pregnancy was known	324 (13.4)	167 (13.2)		327 (14.6)	183 (12.3)	
Alcohol continued in pregnancy	1013 (41.8)	322 (25.5)		991 (44.1)	406 (27.2)	
**Child characteristics**						
Ethnicity, No. (%)			<0.001*			<0.001*
Dutch	1748 (59.9)	583 (40.3)		1647 (59.4)	760	
European	406 (13.9)	310 (21.4)		378 (13.6)	318	
Non-European	762 (26.1)	555 (38.3)		748 (27.0)	650	
Body mass index age 10 years, kg/m^2, median (95% range)	17.1 (14.3, 23.5)	17.0 (14.5, 23.0)	0.768	16.0 (14.4, 22.8)	17.7 (14.6, 23.2)	0.160
Body mass index age 13 years, kg/m^2, median (95% range)	19.6 (15.9, 27.4)	21.3 (16.6, 30.1)	0.051	18.7 (15.6, 25.8)	19.7 (15.7, 29.4)	0.004*

Non-response population includes all Generation R study participants without information on pubertal development outcomes. Values presented as mean (±standard deviation (SD)), median (interquartile range (IQR)) or number of participants valid %). P-values were obtained using the independent sample T-test, the Mann-Whitney U Test or the Chi-square test.
*Statistically significant.

Table S3. Association of birth characteristics with bone health, ovarian/testicular volume at 10 years, and age at menarche, basic model

	Girls			Boys	
**Birth outcomes**	**Difference in relative bone age, months (95% CI)**	**SDS difference in ovarian volume (95% CI)**	**Difference in age at menarche, years (95% CI)**	**Difference in relative bone age, months (95% CI)**	**SDS difference in testicular volume (95% CI)**
**Gestational age at birth**					
Gestational age continuously (per week) [b]	*-0.36 (-0.61, -0.11)*	*-0.01 (-0.03, 0.02)*	*0.02 (-0.01, 0.04)*	*-0.18 (-0.49, 0.13)*	*0.02 (-0.01, 0.04)*
Preterm birth [a]	1.19 (-0.86, 3.25)	0.00 (-0.24, 0.24)	-0.07 (-0.27, 0.13)	-0.84 (-1.83, 3.52)	0.01 (-0.24, 0.25)
Term birth	Reference	Reference	Reference	Reference	Reference
**Birth weight**					
Birth weight continuously (per SD) [b]	*-0.32 (-0.78, 0.13)*	*0.06 (0.00, 0.11)**	*0.08 (0.03, 0.12)**	*0.10 (-0.44, 0.65)*	*0.04 (-0.02, 0.09)*
Birth weight <2500 g [a]	0.55 (-0.56, 2.67)	-0.10 (-0.33, 0.12)	-0.12 (-0.31, 0.07)	2.39 (-0.46, 5.24)	-0.02 (-0.29, 0.25)
Birth weight 2500-4000 g	Reference	Reference	Reference	Reference	Reference
Birth weight >4000 g [a]	0.64 (-0.83, 2.10)	0.14 (-0.03, 0.30)	0.14 (0.02, 0.27)*	1.05 (-0.45, 2.54)	0.15 (0.02, 0.29)*
**Size for gestational age at birth**					
Sex and gestational age adjusted size at birth (per SD) [b]	*-0.12 (-0.56, 0.32)*	*0.06 (0.01, 0.12)**	*0.08 (0.04, 0.13)**	*0.37 (-0.18, 0.92)*	*0.02 (-0.03, 0.07)*
Small (<10th percentile) [a]	1.31 (-0.28, 2.89)	-0.08 (-0.25, 0.10)	-0.14 (-0.28, 0.00)	1.84 (-0.42, 4.10)	0.04 (-0.16, 0.25)
Appropriate (10-90th percentile)	Reference	Reference	Reference	Reference	Reference
Large (>90th percentile) [a]	0.56 (-1.08, 2.19)	0.16 (-0.03, 0.35)	0.15 (0.01, 0.29)*	0.70 (-0.92, 2.19)	0.16 (-0.02, 0.30)

Models include child age at outcome measurement (except for relative bone age). *Statistically significant (Nominal p-value <0.05)

[a] Values represent the change (95% confidence interval (CI)) in relative bone age (months), testicular/ovarian volume (SD) or age at first menstruation (years) for this birthweight category as compared to the reference category.

[b] Values represent the change (95% CI) in relative bone age (months) or testicular/ovarian volume (cm^3) per unit increase.

Table S4. Associations of birth characteristics with pubertal Tanner stage at 13 years, basic model

	Girls		Boys	
**Birth outcome**	**Difference in breast development stage (95% CI)**	**Difference in pubic hair development stage (95% CI)**	**Difference in genital development stage (95% CI)**	**Difference in pubic hair development stage (95% CI)**
**Gestational age at birth**				
Gestational age continuously (per week) [b]	*0.02 (-0.01, 0.04)*	*-0.03 (-0.06, -0.01)*	*-0.01 (-0.03, 0.01)*	*-0.03 (-0.05, 0.00)*
Preterm birth	-0.20 (-0.39, -0.02)	0.00 (-0.22, 0.21)	0.13 (-0.08, 0.33)	0.11 (-0.10, 0.32)
Term birth	Reference	Reference	Reference	Reference
**Birth weight**				
Birth weight continuously (per SD) [b]	*-0.03 (-0.06, 0.01)*	*-0.06 (-0.11, -0.01)*	*-0.02 (-0.07, 0.02)*	*-0.06 (-0.10, -0.01)*
<2500 g [a]	0.07 (-0.12, 0.26)	0.29 (0.06, 0.52)	0.08 (-0.14, 0.30)	0.21 (-0.02, 0.44)
2500-4000 g	Reference	Reference	Reference	Reference
>4000 g [a]	-0.01 (-0.13, 0.11)	0.03 (-0.11, 0.17)	-0.08 (-0.20, 0.04)	-0.07 (-0.19, 0.06)
**Size for gestational age at birth**				
Sex and gestational age adjusted size at birth (per SD) [b]	*-0.05 (-0.08, 0.02)*	*-0.04 (-0.08, 0.00)*	*-0.02 (-0.06, 0.03)*	*-0.05 (-0.09, 0.00)*
Small (<10th percentile) [a]	0.08 (-0.06, 0.22)	0.13 (-0.04, 0.30)	0.07 (-0.11, 0.25)	0.20 (0.02, 0.38)
Appropriate (10-90th percentile)	Reference	Reference	Reference	Reference
Large (>90th percentile) [a]	-0.03 (-0.16, 0.11)	0.00 (-0.16, 0.16)	-0.10 (-0.23, 0.03)	-0.06 (-0.19, 0.07)

Models include child age at outcome measurement. *Statistically significant (FDR adjusted p-value <0.05)

[a] Values represent change (95% confidence interval (CI)) in Tanner Stage for this birthweight category as compared to the reference category.

[b] Values represent change (95% confidence interval (CI)) in Tanner Stage per unit increase

Table S5. Association of infant growth with bone health, ovarian/testicular volume at 10 years and age at menarche, basic model

**Girls**			**Boys**	
**Difference in relative bone age, months (95% CI)**	**SDS difference in ovarian volume (95% CI)**	**Difference in age at menarche, years (95% CI)**	**Difference in relative bone age, months (95% CI)**	**SDS difference in testicular volume (95% CI)**
2.88 (2.47, 3.29)*	0.06 (0.01, 0.10)	-0.12 (-0.16, -0.08)*	3.31 (2.77, 3.86)*	0.09 (0.03, 0.14)*

Results are derived from regression models. Models include child age at outcome measurement (except for relative bone age). *Statistically significant (Nominal p-value <0.05) Values represent the change (95% confidence interval (CI)) in relative bone age (months), testicular/ovarian volume (SD) or age at first menstruation (years) per SD increase in infant growth. Relative bone age was calculated by subtracting the skeletal age from the chronological age.

Table S6. Association of infant growth patterns with pubertal Tanner stage at 13 years, basic model

Girls		Boys	
**Difference in breast development (95% CI)**	**Difference in pubic hair development (95% CI)**	**Difference in genital development (95% CI)**	**Difference in pubic hair development (95% CI)**
0.10 (0.07, 0.14)*	0.08 (0.04, 0.13)*	0.04 (-0.01, 0.09)	0.11 (0.06, 0.16)*

Results are derived from regression models including all fetal and infant growth patterns. Values represent change (95% confidence interval (CI)) in Tanner Stage per SD increase in infant growth.

Table S7. Association of fetal and infant growth patterns with pubertal Tanner stage at 13 years, adjusted model

		**Girls**		**Boys**	
		**Difference in breast development (95% CI)**	**Difference in pubic hair development (95% CI)**	**Difference in genital development (95% CI)**	**Difference in pubic hair development (95% CI)**
**Fetal growth**	**Infant growth**	**OR (95% CI)**	**OR (95% CI)**	**OR (95% CI)**	**OR (95% CI)**
Deceleration	Deceleration	-0.10 (-0.26, 0.05)	-0.08 (-0.27, 0.10)	-0.04 (-0.22, 0.15)	-0.07 (-0.25, 0.11)
	Normal	0.09 (-0.06, 0.24)	-0.04 (-0.22, 0.13)	-0.02 (-0.21, 0.16)	0.00 (-0.19, 0.19)
	Acceleration	0.17 (-0.06, 0.41)	0.05 (-0.23, 0.33)	0.14 (-0.19, 0.47)	-0.08 (-0.39, 0.24)
Normal	Deceleration	0.00 (-0.17, 0.16)	-0.12 (-0.32, 0.08)	-0.17 (-0.37, 0.03)	-0.28 (-0.49, -0.08)*
	Normal	*Reference*	*Reference*	*Reference*	*Reference*
	Acceleration	0.21 (0.06, 0.37)*	0.11 (-0.08, 0.30)	-0.05 (-0.26, 0.16)	0.13 (-0.08, 0.33)
Acceleration	Deceleration	-0.15 (-0.40, 0.10)	-0.10 (-0.38, 0.19)	-0.03 (-0.35, 0.30)	-0.30 (-0.63, 0.03)
	Normal	0.07 (-0.09, 0.23)	0.06 (-0.14, 0.25)	-0.02 (-0.21, 0.18)	0.17 (-0.03, 0.37)
	Acceleration	0.13 (-0.03, 0.29)	0.13 (-0.06, 0.31)	-0.12 (-0.32, 0.08)	-0.05 (-0.25, 0.16)

Results are derived from regression models including all fetal and infant growth patterns. Models include maternal age, body-mass index, educational level, ethnicity, folic acid supplementation use, smoking and alcohol use in pregnancy, and child age at outcome measurement. Values represent change (95% confidence interval (CI)) in Tanner Stage for the fetal or infant growth categories as compared to normal growth category, from conditional models. P-values are Federal Discovery Rate adjusted.

Table S8. Association of birth characteristics with bone health, ovarian/testicular volume at 10 years, and age at menarche, adjusted model. Sensitivity analysis including Dutch children only.

	Girls			Boys	
**Birth outcomes**	**Difference in relative bone age, months (95% CI)**	**SDS difference in ovarian volume (95% CI)**	**Difference in age at menarche, years (95% CI)**	**Difference in relative bone age, months (95% CI)**	**SDS difference in testicular volume (95% CI)**
**Gestational age at birth**					
Gestational age continuously (per week) [b]	*-0.16 (-0.46, 0.14)*	*0.01 (-0.03, 0.04)*	*0.01 (-0.02, 0.04)*	*0.03 (-0.38, 0.44)*	*0.02 (-0.02, 0.05)*
Preterm birth [a]	0.50 (-2.05, 3.06)	-0.03 (-0.35, 0.30)	-0.06 (-0.28, 0.17)	-0.85 (-4.43, 2.74)	0.19 (-0.12, 0.49)
Term birth	Reference	Reference	Reference	Reference	Reference
**Birth weight**					
Birthweight continuously (per SD) [b]	*0.15 (-0.39, 0.69)*	*0.08 (0.02, 0.14)**	*0.06 (0.00, 0.11)*	*0.58 (-0.14, 1.28)*	*0.05 (-0.02, 0.11)*
Birthweight <2500 g [a]	0.46 (-2.33, 3.25)	-0.06 (-0.38, 0.26)	-0.17 (-0.41, 0.07)	0.24 (-3.65, 4.12)	0.13 (-0.21, 0.47)
Birthweight 2500-4000 g	Reference	Reference	Reference	Reference	Reference
Birthweight >4000 g [a]	0.69 (-0.93, 2.30)	0.09 (-0.10, 0.28)	0.05 (-0.04, 0.22)	1.01 (-0.78, 2.79)	0.25 (0.09, 0.40)*
**Size for gestational age at birth**					
Sex and gestational age adjusted size at birth (per SD) [b]	*0.31 (-0.23, 0.84)*	*0.08 (0.02, 0.15)**	*0.06 (0.01, 0.11)*	*0.64 (-0.07, 1.34)*	*0.04 (-0.02, 0.10)*
Small (<10th percentile) [a]	0.06 (-1.99, 2.10)	-0.11 (-0.35, 0.13)	-0.14 (-0.32, 0.05)	0.59 (-2.41, 3.59)	0.20 (-0.06, 0.45)
Appropriate (10-90th percentile)	Reference	Reference	Reference	Reference	Reference
Large (>90th percentile) [a]	0.80 (-0.98, 2.59)	0.11 (-0.09, 0.31)	0.08 (-0.07, 0.23)	0.48 (-1.43, 2.39)	0.25 (0.09, 0.41)*

Models include maternal age, body-mass index, educational level, ethnicity, folic acid supplementation use, smoking and alcohol use in pregnancy, and child age at outcome measurement (except for relative bone age). *Statistically significant (FDR adjusted p-value <0.05)

[a] Values represent the change (95% confidence interval (CI)) in relative bone age (months), testicular/ovarian volume (SD) or age at menarche (years) for this birthweight category as compared to the reference category.

[b] Values represent the change (95% CI) in relative bone age (months), testicular/ovarian volume (cm^3) or age at menarche (years) per unit increase.

Table S9. Association of birth characteristics with pubertal Tanner stage at 13 years, adjusted model. Sensitivity analysis including Dutch children only.

	**Girls**		**Boys**	
**Birth outcome**	**Difference in breast development stage (95% CI)**	**Difference in pubic hair development stage (95% CI)**	**Difference in genital development stage (95% CI)**	**Difference in pubic hair development stage (95% CI)**
**Gestational age at birth**				
Gestational age continuously (per week) [b]	*0.02 (-0.01, 0.05)*	*-0.04 (-0.07, 0.00)*	*-0.03 (-0.06, 0.00)*	*-0.02 (-0.05, 0.01)*
Preterm birth	-0.12 (-0.36, 0.12)	-0.02 (-0.30, 0.26)	0.26 (-0.02, 0.54)	0.06 (-0.24, 0.36)
Term birth	Reference	Reference	Reference	Reference
**Birth weight**				
Birth weight continuously (per SD) [b]	*0.00 (-0.05, 0.05)*	*-0.05 (-0.11, 0.01)*	*-0.04 (-0.10, 0.02)*	*-0.03 (-0.09, 0.03)*
<2500 g [a]	0.18 (-0.09, 0.45)	0.33 (-0.01, 0.66)	0.17 (-0.13, 0.46)	0.10 (-0.21, 0.41)
2500-4000 g	Reference	Reference	Reference	Reference
>4000 g [a]	0.10 (-0.04, 0.25)	0.06 (-0.11, 0.23)	-0.07 (-0.21, 0.08)	-0.01 (-0.16, 0.14)
**Size for gestational age at birth**				
Sex and gestational age adjusted size at birth (per SD) [b]	*-0.03 (-0.07, 0.02)*	*-0.03 (-0.08, 0.03)*	*-0.01 (-0.07, 0.05)*	*-0.01 (-0.07, 0.05)*
Small (<10th percentile) [a]	0.08 (-0.12, 0.28)	0.19 (-0.04, 0.42)	0.11 (-0.13, 0.36)	0.10 (-0.15, 0.34)
Appropriate (10-90th percentile)	Reference	Reference	Reference	Reference
Large (>90th percentile) [a]	0.07 (-0.09, 0.23)	0.05 (-0.14, 0.23)	-0.08 (-0.23, 0.08)	-0.03 (-0.13, 0.18)

Models include maternal age, body-mass index, educational level, ethnicity, folic acid supplementation use, smoking and alcohol use in pregnancy, and child age at outcome measurement. *Statistically significant (FDR adjusted p-value <0.05)

[a] Values represent change (95% confidence interval (CI)) in Tanner Stage for this birthweight category as compared to the reference category.

[b] Values represent change (95% confidence interval (CI)) in Tanner Stage per unit increase

Table S10. Association of infant growth with bone health, ovarian/testicular volume at 10 years and age at menarche, adjusted model. Sensitivity analysis including Dutch children only.

**Girls**			**Boys**	
**Difference in relative bone age, months (95% CI)**	**SDS difference in ovarian volume (95% CI)**	**Difference in age at menarche, years (95% CI)**	**Difference in relative bone age, months (95% CI)**	**SDS difference in testicular volume (95% CI)**
2.17 (1.65, 2.69)	0.03 (-0.03, 0.08)	-0.10 (-0.15, 0.05)	2.82 (2.11, 3.52)*	0.08 (0.02, 0.13)*

Results are derived from regression models including all fetal and infant growth patterns. Models include maternal age, body-mass index, educational level, ethnicity, folic acid supplementation use, smoking and alcohol use in pregnancy, and child age at outcome measurement (except for relative bone age). *Statistically significant (FDR adjusted p-value <0.05). Values represent the change (95% confidence interval (CI)) in relative bone age (months), testicular/ovarian volume (SD) or age at first menstruation (years) per SD increase in infant growth.

Table S11. Association of fetal and infant growth patterns with pubertal Tanner stage at 13 years, adjusted model. Sensitivity analysis including Dutch children only

**Girls**		**Boys**	
**Difference in breast development (95% CI)**	**Difference in pubic hair development (95% CI)**	**Difference in genital development (95% CI)**	**Difference in pubic hair development (95% CI)**
0.07 (0.02, 0.12)*	0.06 (0.01, 0.12)*	0.03 (-0.03, 0.09)	0.09 (0.02, 0.15)*

Results are derived from regression models including all fetal and infant growth patterns. Models include maternal age, body-mass index, educational level, ethnicity, folic acid supplementation use, smoking and alcohol use in pregnancy, and child age at outcome measurement. Values represent change (95% confidence interval (CI)) in Tanner Stage per SD increase in infant growth.

Figure S1. Flowchart of participants included in the study.

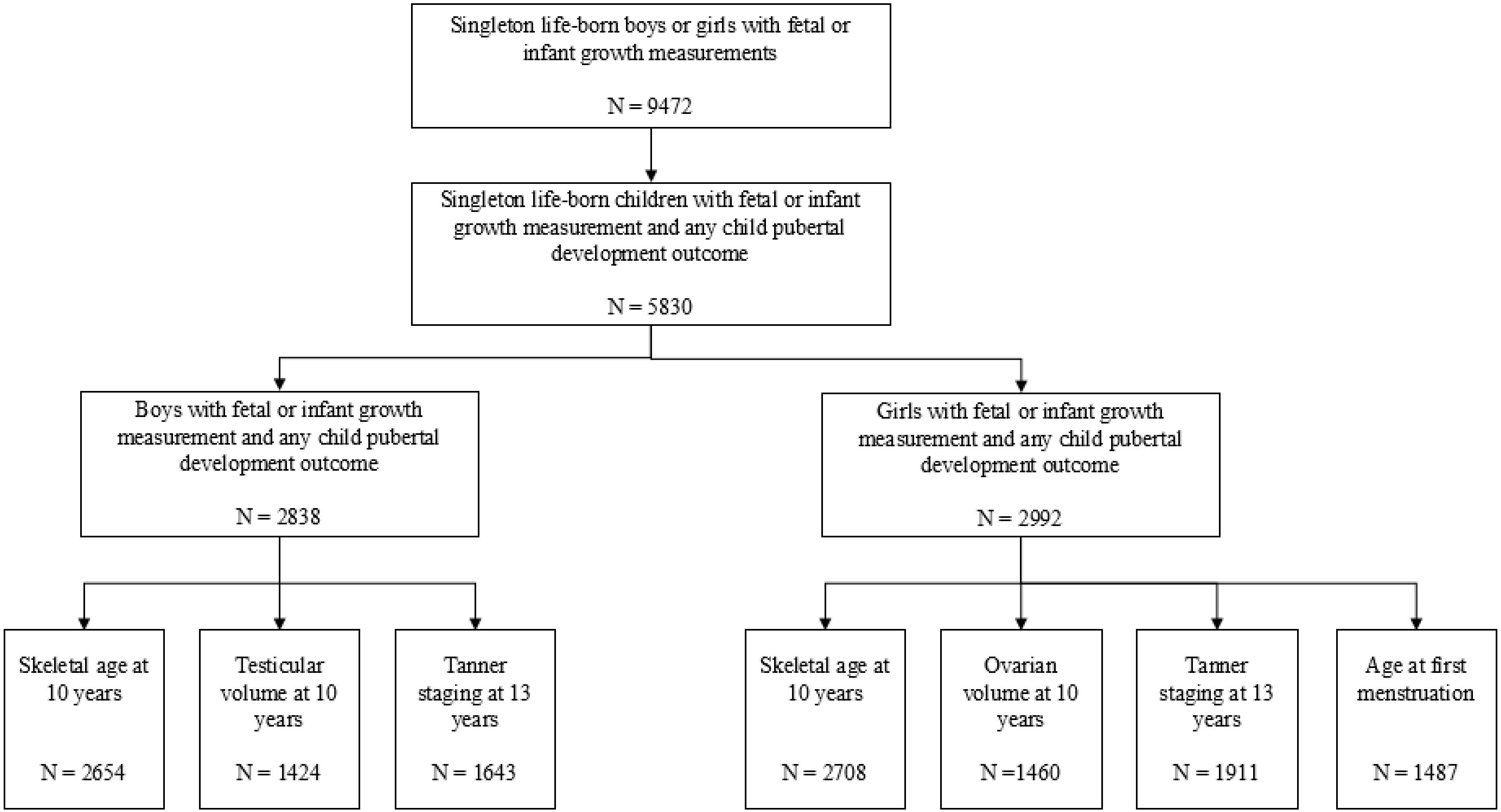

Figure S2. Directed Acyclic Graph.

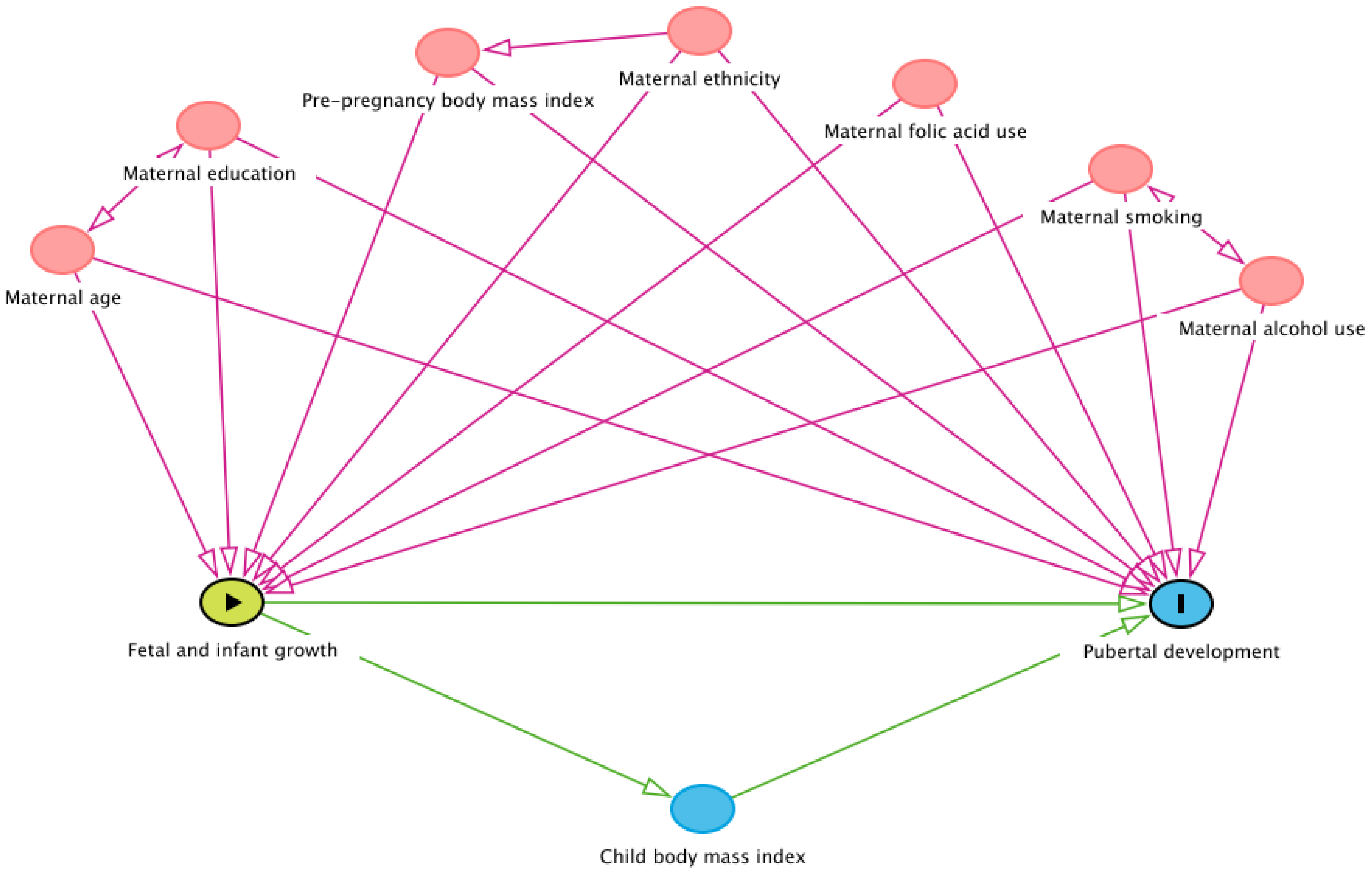

Figure S3. Plot on the correlation between the pubertal development outcomes for A) girls and B) boys

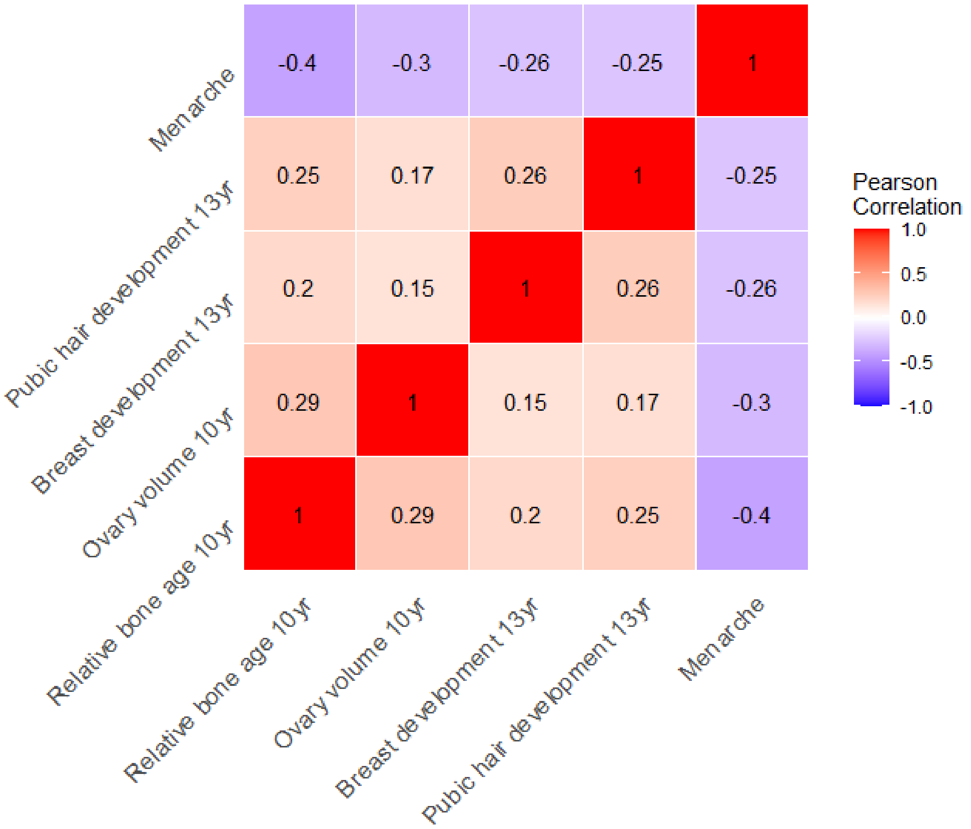

A)

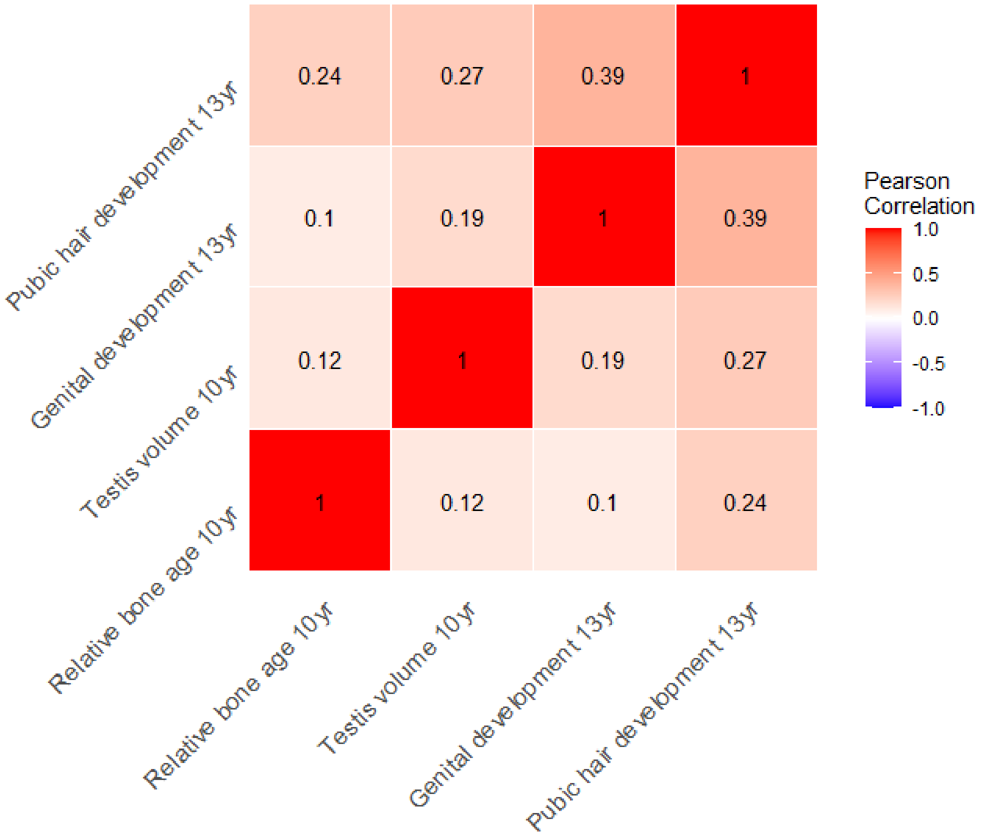

B)
